# Supplementary material for: Manipulation of nonlinear optical responses in layered ferroelectric niobium oxide dihalides
Source: Nat Commun. 2023 Sep 22;14:5911. doi: 10.1038/s41467-023-41383-7 (PMC10516934; doi:10.1038/s41467-023-41383-7)
Supplement: Supplementary file 1 — Supplemental Information [file 41467_2023_41383_MOESM1_ESM.pdf]

Supplementary Information

Manipulation of Nonlinear Optical Responses in Layered Ferroelectric Niobium Oxide Dihalides

This PDF file includes:

Supplementary Fig. 1: Metal-to-insulator phase transition.

Supplementary Fig. 2: Band structures for $NbOCl_2$ with different layer thicknesses.

Supplementary Fig. 3: Symmetry selection rules for bulk and monolayer $NbOCl_2$.

Supplementary Fig. 4: Five independent shift current susceptibility components in monolayer $NbOCl_2$.

Supplementary Fig. 5: Band structures for monolayer $NbOCl_2$ under different uniaxial strains.

Supplementary Fig. 6: The (0, *k*, *l*) reflections of $NbOCl_2$ obtained by single crystal X-ray diffraction.

Supplementary Fig. 7: The (0, *k*, *l*) reflections of $NbOI_2$ obtained by single crystal X-ray diffraction.

Supplementary Fig. 8: Normalized SHG intensity of single-crystal $NbOCl_2$ as a function of temperature.

Supplementary Fig. 9: Total density of states (TDOS) of the bulk $NbOCl_2$ under different pressures (*left* panel) and temperatures (*right* panel).

Supplementary Fig. 10: Nonlinear optical responses for monolayer $NbOX_2$ (X = Cl, Br, I).

Supplementary Fig. 11: Band structures with scissor operators for $NbOX_2$ (X = Cl, Br, I).

Supplementary Fig. 12: SHG Responses in $NbOBr_2$.

Supplementary Fig. 13: SHG Responses in $NbOI_2$.

Supplementary Fig. 14: Convergence tests on the density of k-grid for NLO responses.

Supplementary Fig. 15: XRD of the synthesized single crystal $NbOI_2$.

Supplementary Note 1: Symmetry selection rules in bulk and monolayer $NbOCl_2$.

Supplementary Note 2: Expand of the generalized derivative of the dipole matrix element by using sum rule.

Supplementary Tables 1-21: Structure information of bulk $NbOCl_2$ and $NbOI_2$ by using single-crystal X-ray diffraction analysis.

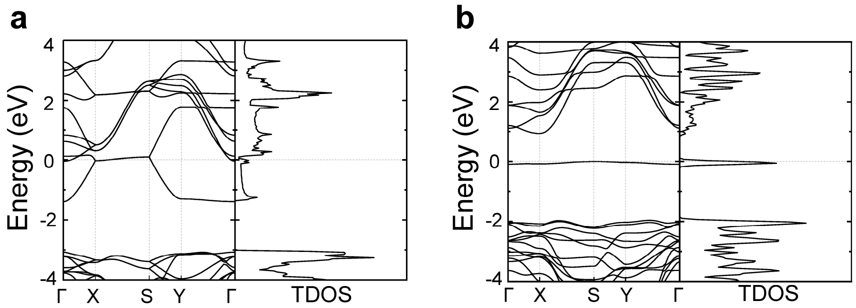

Supplementary Fig. 1 | Metal-to-insulator phase transition. a Band structure and total density of states (DOS) of monolayer $NbOCl_2$ without Peierls distortion, showing a metallic feature. **b** Same as **a** but for monolayer $NbOCl_2$ with the Peierls distortion formed by Nb atoms, showing an insulating feature. Fermi level is set to zero.

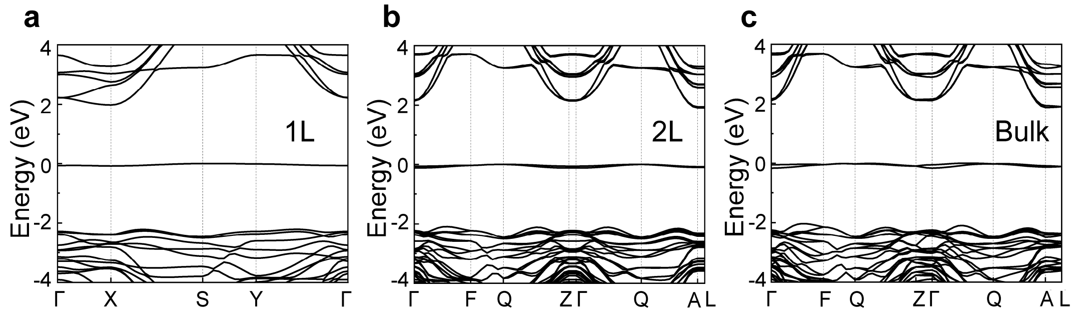

Supplementary Fig. 2 | Band structures for $NbOCl_2$ with different layer thicknesses. **a-c** HSE-calculated band structures for monolayer $NbOCl_2$, bilayer $NbOCl_2$ and bulk $NbOCl_2$, which have the indirect bandgaps of ~1.98 eV, ~1.91 eV and ~1.88 eV, respectively. Similar bandgaps indicate the weak interlayer coupling in $NbOCl_2$. Fermi level is set to zero.

a

b

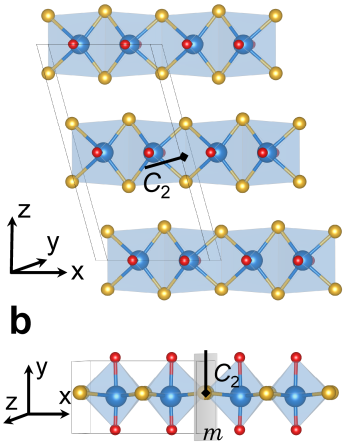

c

C_2					
0	0	0	xyz	0	xxy
yxx	yyy	yzz	0	yxz	0
0	0	0	zyz	0	zxy

d

C_{2V}					
0	0	0	0	0	xxy
yxx	yyy	yzz	0	0	0
0	0	0	zyz	0	0

Supplementary Fig. 3 | Symmetry selection rules for bulk and monolayer $NbOCl_2$. a, b Crystal structures of bulk and monolayer $NbOCl_2$, respectively. Here, x, y, z represents the cartesian axes. Here, C_2 implies 2-fold rotation operation and m is mirror plane. **c, d** The SHG susceptibility tensor $\chi^{(2)}(2\omega;\omega,\omega)$ of monoclinic C_2 point group and orthorhombic C_{2v} point group.

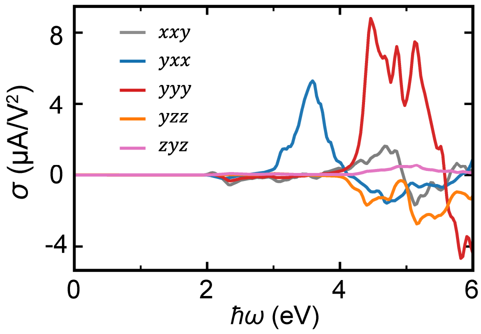

Supplementary Fig. 4 | Five independent shift current susceptibility components in monolayer $NbOCl_2$.

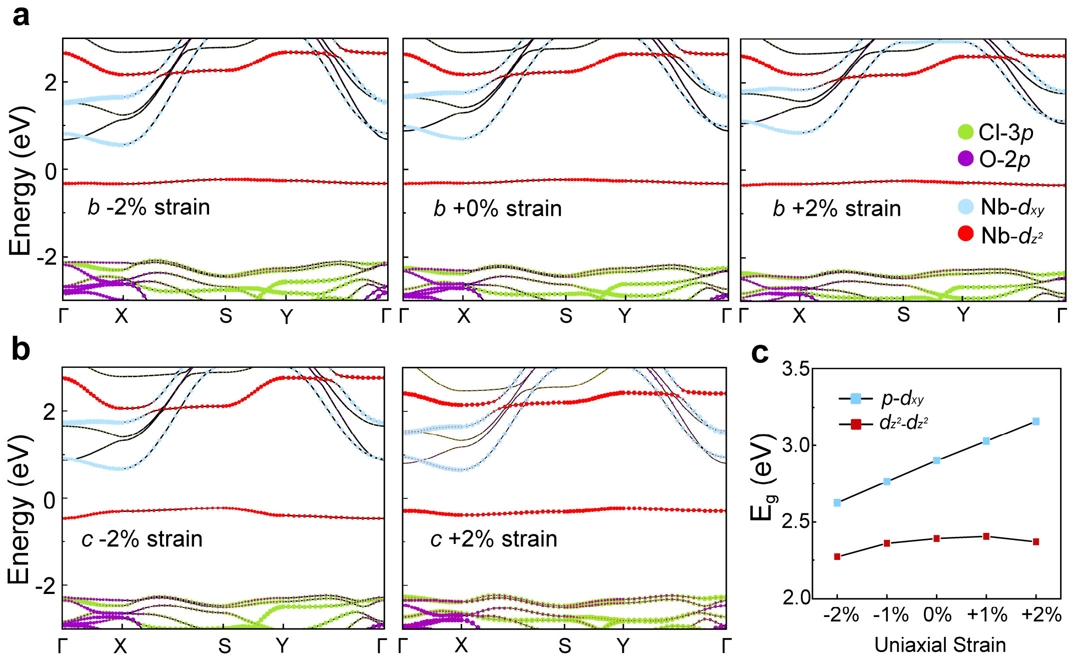

Supplementary Fig. 5 | Band structures for monolayer $NbOCl_2$ under different uniaxial strains. **a** Orbital-projected band structures via applying -2%, 0, and 2% uniaxial strain along *b* direction. **b** Band structures via applying -2% and 2% uniaxial strain along ***c*** direction. Fermi level is set to zero. **c** Energy bandgap as a function of uniaxial strain. Here, blue dots represent the energy gap between Cl *p* orbital and Nb d_{xy} orbital under the strain along *b* direction, and red dots represent the energy gap between two flat Nb d_{z2} orbitals under the strain along *c* direction.

The strain along *b* direction mainly changes the length of Nb-O bond and further influences the energy level repulsion between conduction band contributed by Nb atoms and valance bands contributed by anion. Differently, the strain along *c* direction changes the strength of Peierls distortion and only changes the dispersion of Nb d_{z2} orbitals. Therefore, as shown in **c**, the energy gap between two Nb d_{z2} states is insensitive to the in-plane strain. However, the energy gap between *p* and *d* states increases when the strain along *b* direction changes from -2 % to 2 %.

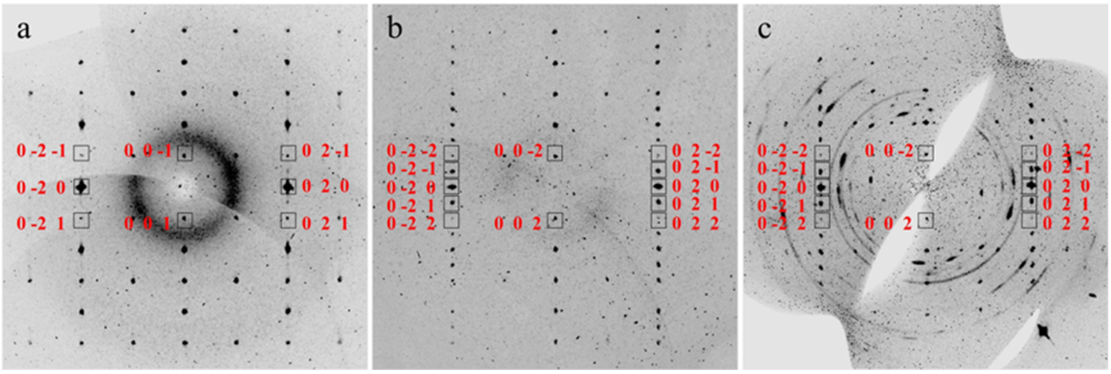

Supplementary Fig. 6 | The (0, *k*, *l*) reflections of $NbOCl_2$ obtained by single crystal X-ray diffraction. a Diffraction patterns collected at ambient condition. **b** Diffraction patterns collected at 500 K. **c** Diffraction patterns collected at 500 K.

Bragg diffraction patterns in (0, *k*, *l*) are marked by their corresponding indices. Under high temperature or pressure, additional reflections along the (0, ±2, *l*) lines appear (Supplementary Fig. 6b and 6c), *i.e.*, the miller indices (0, *k*, *l*) regarded to the structure at ambient condition is transformed to (0, *k*, *2l*) under high temperature or pressure. Thus, the unit cell will be doubled along ***c*** axis when increasing temperature or applying pressure, which is consistent with the unique AFE phase reported here because the ↑↑↓↓ polarization arrangement just requires the doubled unit cell along *c* axis of the FE phase.

The detailed structural information can be obtained by fitting results on the original diffraction pattern (see Method section in the main text). At the ambient condition, we list two possible unit cells with different Z values which are defined as numbers of formulas per unit cell (Supplementary Table 1). $NbOCl_2$ prefers the structure with Z=4 instead of Z=8 (Z=4: R=0.034, wR=0.077; Z=8: R=0.057, wR=0.109) after performing the structural refinements, with weak reflections caused by structural distortion, which is consistent with the structure reported in previous literature [1,2]. Under high temperature or high pressure, however, the extra reflections suggest the doubled Z value of the crystal structures, implying the structural phase transitions of $NbOCl_2$. As shown in Supplementary Tables 2 and 17, the fitting results after performing the structural refinements show that the structures under high temperature (500 K, R=0.078, wR=0.160) or pressure (5.7 GPa, R=0.089, wR=0.227) prefer Z=8.

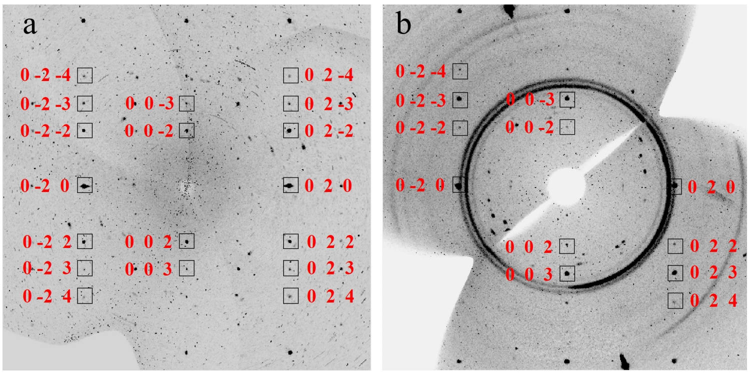

Supplementary Fig. 7 | The (0, *k*, *l*) reflections of $NbOI_2$ obtained by single crystal X-ray diffraction. **a** Diffraction patterns collected at ambient condition. **b** Diffraction patterns collected at 10.7 GPa.

$NbOI_2$ share the same symmetry at ambient condition like $NbOCl_2$, but some weak reflections, e.g. (0, ±2, ±1), are barely seen in Supplementary Fig. 7a. Some important structural information caused by the inconspicuous changes in diffraction patterns, e.g., the changes of the reflection intensity of (0, 0, 3), can only be refined by fitting results on the original diffraction pattern. The fitting results under 10.7 GPa (Supplementary Tables 7 and 12) support a change from a non-centrosymmetric structure with *C*2 space group to a centrosymmetric structure with *C*2/*m* space group, which is also consistent with the SHG measurements (see Fig. 4e in main text).

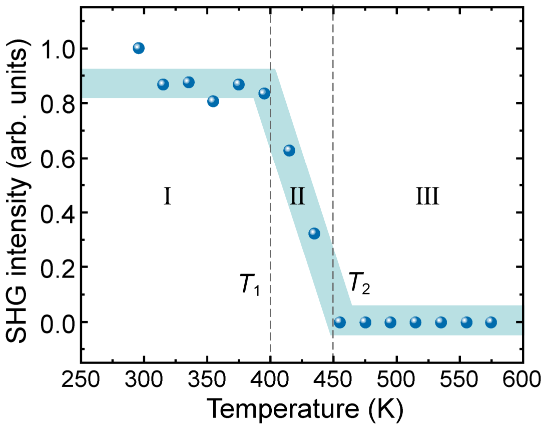

Supplementary Fig. 8 | Normalized SHG intensity of single-crystal $NbOCl_2$ as a function of temperature. The SHG intensity also undergoes three similar stages like under pressure, i.e., from FE phase (stage I) to FE-AFE mixing phase (stage II) and to AFE phase (stage III). The normalized SHG intensity starts to decrease at T_1~400 K and eventually reaches zero at T_2~450 K.

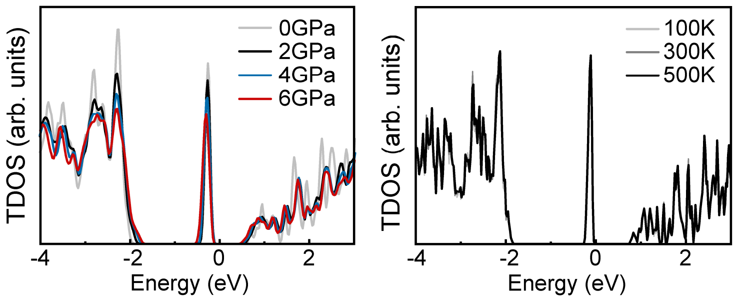

Supplementary Fig. 9 | Total density of states (TDOS) of the bulk $NbOCl_2$ under different pressures (*left* panel) and temperatures (*right* panel). The change of crystal lattice caused by pressure and temperature brings about similar TDOS with moving band edge slightly, implying that SHG spectra will change continuously or regularly under pressure and temperature. However, in the SHG measurement, the response keeps an average level under low pressure and diminish dramatically to zero over a certain pressure as shown in Fig. 4d. Hence, the speculation, SHG intensity diminish dramatically due to the shift of the edge for different temperature or pressure, could be ruled out.

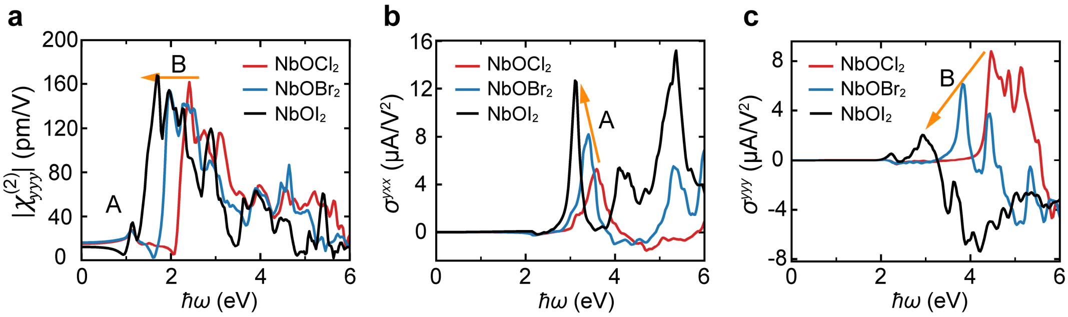

Supplementary Fig. 10 | Nonlinear optical responses for monolayer $NbOX_2$ (X = Cl, Br, I). a Calculated SHG susceptibility $\left|\chi_{yyy}^{(2)}\right|$ as a function of incident photon energy. **b-c** Frequency-dependent shift current susceptibility tensors σ^{yxx} and σ^{yyy}. For $NbOX_2$, SHG strengths for peak B are less changed for different X. However, the peaks of σ show different changes for different susceptibility tensor components with varying X from Cl to I.

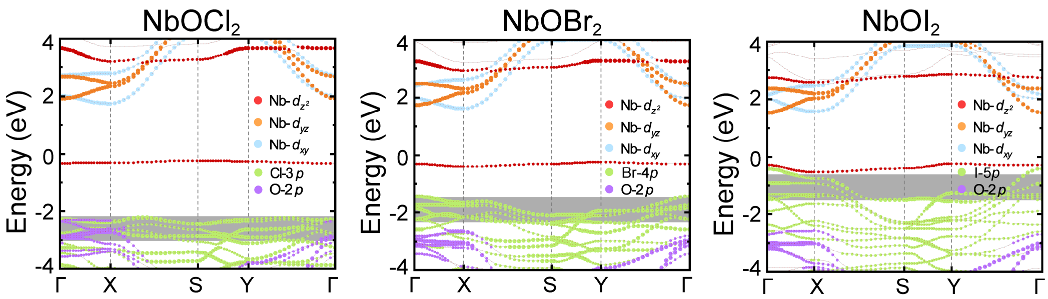

Supplementary Fig. 11 | Band structures with scissor operators for NbOX_2 (X = Cl, Br, I). Fermi level is set to zero. The grey shadows show the orbitals which contribute the SHG peaks of three materials. The double-photons resonance in $NbOI_2$ and $NbOBr_2$ comes from X p → Nb d, while O p orbitals also contribute in $NbOCl_2$ (X p + O p → Nb d).

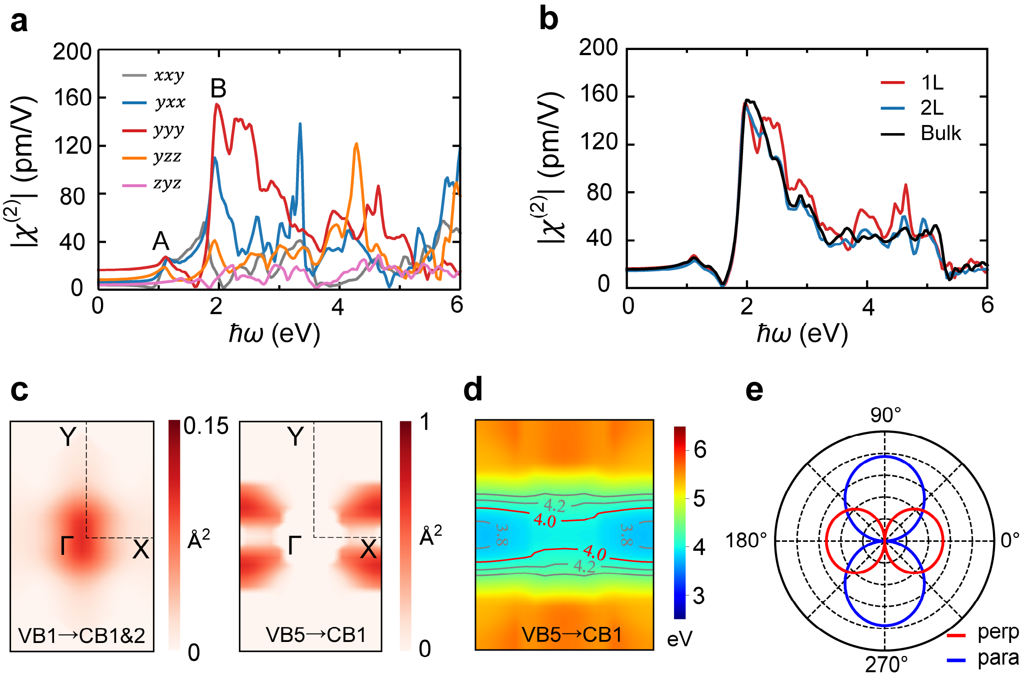

Supplementary Fig. 12 | SHG Responses in $NbOBr_2$. a Calculated nonlinear susceptibility $|\chi^{(2)}_{abc}|$ of monolayer $NbOBr_2$ as a function of incident photon energy. **b** SHG response for monolayer, bilayer, and bulk $NbOBr_2$. **c** k-resolved absorption strength, $r^y_{mn}r^y_{nm}$ (Å^2), occurred near A and B peaks in SHG spectrum. Here, VB_n (CB_n) labels the nth valance (conduction) band starting from the top valance (bottom conduction) band. **d** Contour plot of energy difference of specified two bands. **e** Angle-resolved SHG polarization. Here, the photon energy of the incident light is set to be 1.96 eV, and the red (blue) curve represents the response with the direction perpendicular (parallel) to the polarization of incident light.

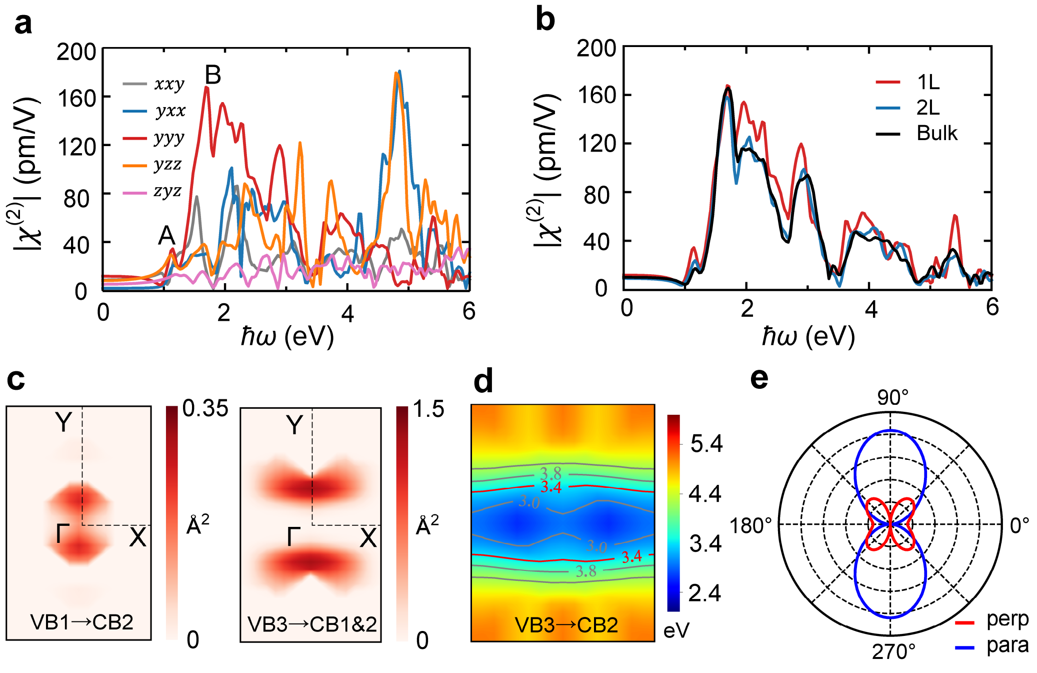

Supplementary Fig. 13 | SHG Responses in $NbOI_2$. **a** Calculated nonlinear susceptibility $|\chi^{(2)}_{abc}|$ of monolayer $NbOI_2$ as a function of incident photon energy. **b** SHG response for monolayer, bilayer, and bulk $NbOI_2$. **c** *k*-resolved absorption strength, $r^y_{mn}r^y_{nm}$ (Å^2), occurred near A and B peaks in SHG spectrum. Here, VB_n (CB_n) labels the nth valance (conduction) band starting from the top valance (bottom conduction) band. **d** Contour plot of energy difference of specified two bands. **e** Angle-resolved SHG polarization. Here, the photon energy of the incident light is set to be 1.69 eV, and the red (blue) curve represents the response with the direction perpendicular (parallel) to the polarization of incident light.

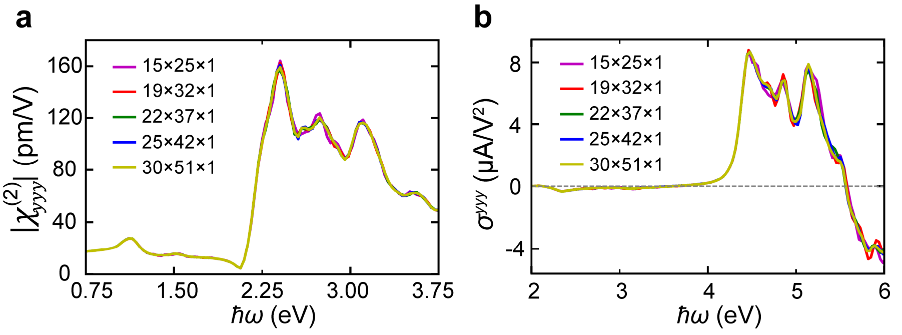

Supplementary Fig. 14 | Convergence tests on the density of *k*-grid for NLO responses. a Convergence test for the SHG susceptibility $\left|\chi^{(2)}_{yyy}\right|$ of monolayer $NbOCl_2$. **b** Convergence test for the shift current susceptibility σ^{yyy} of monolayer $NbOCl_2$.

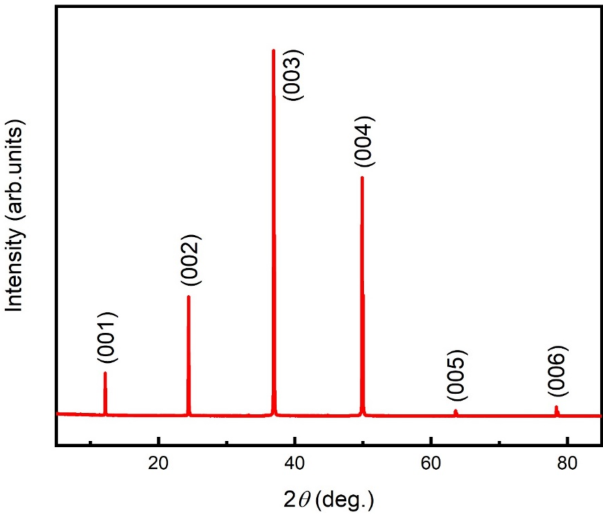

Supplementary Fig. 15 | XRD of the synthesized single crystal $NbOI_2$.

Supplementary Note 1. Symmetry selection rules in bulk and monolayer $NbOCl_2$.

Monoclinic C_2 symmetry and orthorhombic C_{2v} symmetry have partially uniform symmetry selection rules due to the double rotational symmetry. To see this point clearly, we plot the crystal structures of bulk $NbOCl_2$ and monolayer $NbOCl_2$ in Supplementary Fig. 3, where the corresponding coordinate systems are displayed. The second-order susceptibility tensor $\chi^{(2)}_{abc}$ (a, b, c represents cartesian coordinates) has 27 components. The spatial symmetry causes $\chi^{(2)}_{abc} = T_{aa'}T_{bb'}T_{cc'}\chi^{(2)}_{a'b'c'}$, where T is transformation matrix, and the right side of above equation satisfies the Einstein's summation rule. For the monoclinic C_2 symmetry, there is a double rotational symmetry about y axis whose transformation matrix can be expressed as

$$T = \begin{pmatrix} -1 & 0 & 0 \\ 0 & 1 & 0 \\ 0 & 0 & -1 \end{pmatrix}.$$

When the left side of above equation walk through all elements, we can list 27 equations. Considering intrinsic permutation symmetry, there is a non-zero component distribution of 3×6 matrix shown in Supplementary Fig. 3c. On the other hand, for the orthorhombic C_{2v} symmetry, there is an additional mirror symmetry about the yOz plane which satisfies

$$T = \begin{pmatrix} -1 & 0 & 0 \\ 0 & 1 & 0 \\ 0 & 0 & 1 \end{pmatrix},$$

which eliminates three components: xyz, yxz, and zxy, resulting in the 3×6 matrix shown in Supplementary Fig. 3d. Comparing Supplementary Fig. 3c and Supplementary Fig. 3d, it can be found that some non-zero components of the second-order susceptibility tensor are all surviving.

Due to the extremely weak interlayer interaction of $NbOCl_2$, stacking multiple layers have similar optical response as monolayer (see Fig. 2f in the main text). Therefore, the SHG simulations performed on monolayer $NbOCl_2$ are comparable to monoclinic bulk $NbOCl_2$.

Supplementary Note 2. Expand of the generalized derivative of the dipole matrix element by using sum rule.

The generalized derivative of the dipole matrix element was expressed as in geometric form,

$$r^a_{nm;b}(\boldsymbol{k}) \equiv \frac{\partial r^a_{nm}(\boldsymbol{k})}{\partial k^b} - i[\xi^b_{nn}(\boldsymbol{k}) - \xi^b_{mm}(\boldsymbol{k})]r^a_{nm}(\boldsymbol{k}). \tag{1}$$

Here, a, b indicate Cartesian components, $\xi^a_{nm} = i\int dx u^*_n(\boldsymbol{k},\boldsymbol{x})\frac{\partial}{\partial k^a}u_m(\boldsymbol{k},\boldsymbol{x})$, which is called Berry connection when $n = m$, and $r^a_{nm}(\boldsymbol{k}) \equiv \xi^a_{nm}(\boldsymbol{k})$ $(n \neq m)$ is non-Abelian Berry connection. Equation (1) could be expanded in detail by using the commutation relation $[r^a, p^b] = i\hbar\delta^{ab}$. We start from the commutation with Bloch states $|n\boldsymbol{k}\rangle$ labeled by a band index n and crystal momentum $\boldsymbol{k}$,

$$\begin{aligned}\left\langle n\boldsymbol{k}\middle|[r^a,p^b]\middle|m\boldsymbol{k}'\right\rangle &= \left\langle n\boldsymbol{k}\middle|[r^a_i,p^b]\middle|m\boldsymbol{k}'\right\rangle + \left\langle n\boldsymbol{k}\middle|[r^a_e,p^b]\middle|m\boldsymbol{k}'\right\rangle \\ &= i\hbar\delta^{ab}\delta_{nm}\delta\left(\boldsymbol{k}-\boldsymbol{k}'\right).\end{aligned} \tag{2}$$

When $n \neq m$, the intraband part can be expressed as

$$\begin{aligned}\left\langle n\boldsymbol{k}\middle|[r^a_i,p^b]\middle|m\boldsymbol{k}'\right\rangle &= i\delta\left(\boldsymbol{k}-\boldsymbol{k}'\right)(p^b_{nm})_{;k^a} = i\delta\left(\boldsymbol{k}-\boldsymbol{k}'\right)(im\omega_{nm}r^b_{nm})_{;k^a} \\ &= -m\delta\left(\boldsymbol{k}-\boldsymbol{k}'\right)\omega_{nm}(r^b_{nm})_{;k^a} - m\delta\left(\boldsymbol{k}-\boldsymbol{k}'\right)r^b_{nm}\Delta^a_{nm},\end{aligned} \tag{3}$$

which use a relation involving intraband position operator $\mathbf{r_i}$ and simple operator $\boldsymbol{S}$,

$$\left\langle n\boldsymbol{k}\middle|[\boldsymbol{r_i},\boldsymbol{S}]\middle|m\boldsymbol{k}'\right\rangle = i\delta\left(\boldsymbol{k}-\boldsymbol{k}'\right)(\boldsymbol{S}_{nm})_{;\boldsymbol{k}},\ (\boldsymbol{S}_{nm})_{;\boldsymbol{k}} \equiv \frac{\partial \boldsymbol{S}_{nm}}{\partial \boldsymbol{k}} - i\boldsymbol{S}_{nm}(\boldsymbol{\xi}_{nn} - \boldsymbol{\xi}_{mm}).$$

The simple operator $\boldsymbol{S}$ is defined as one whose Bloch state matrix elements involve only $\delta\left(\boldsymbol{k}-\boldsymbol{k}'\right)$, i.e., $\left\langle n\boldsymbol{k}\middle|\boldsymbol{S}\middle|m\boldsymbol{k}'\right\rangle = \delta\left(\boldsymbol{k}-\boldsymbol{k}'\right)\boldsymbol{S}_{nm}$.

The interband part can be expanded by inserting normalized complete basis,

$$\begin{aligned}\left\langle n\boldsymbol{k}\middle|[r^a_e,p^b]\middle|m\boldsymbol{k}'\right\rangle &= \sum_{l,\boldsymbol{k_t}}\langle n\boldsymbol{k}|r^a_e|l\boldsymbol{k_t}\rangle\left\langle l\boldsymbol{k_t}\middle|p^b\middle|m\boldsymbol{k}'\right\rangle - \langle n\boldsymbol{k}|p^b|l\boldsymbol{k_t}\rangle\left\langle l\boldsymbol{k_t}\middle|r^a_e\middle|m\boldsymbol{k}'\right\rangle \\ &= \sum_{l,\boldsymbol{k_t}}(1-\delta_{nl})\,\delta(\boldsymbol{k}-\boldsymbol{k_t})\xi^a_{nl}\cdot p^b_{lm}\delta\left(\boldsymbol{k_t}-\boldsymbol{k}'\right) \\ &\quad -\sum_{l,\boldsymbol{k_t}}p^b_{nl}\delta(\boldsymbol{k}-\boldsymbol{k_t})\cdot(1-\delta_{lm})\,\delta\left(\boldsymbol{k_t}-\boldsymbol{k}'\right)\xi^a_{lm} \\ &= \sum_{l,l\neq m\neq n}\delta\left(\boldsymbol{k}-\boldsymbol{k}'\right)(\xi^a_{nl}\cdot p^b_{lm} - \xi^a_{lm}\cdot p^b_{nl}) \\ &\quad +\delta\left(\boldsymbol{k}-\boldsymbol{k}'\right)(\xi^a_{nm}\cdot p^b_{mm} - \xi^a_{nm}\cdot p^b_{nn}) \\ &= m\delta\left(\boldsymbol{k}-\boldsymbol{k}'\right)\left(r^a_{nm}\Delta^b_{mn} + i\sum_{l,l\neq m\neq n}\left(\omega_{lm}r^a_{nl}r^b_{lm} - \omega_{nl}r^a_{lm}r^b_{nl}\right)\right).\end{aligned} \tag{4}$$

Here, $\boldsymbol{p}_{nm} = im\boldsymbol{r}_{nm}\omega_{nm}$, ω_{nm} is the energy difference of the nth band and m^{th} band. By using equation (1): $\left\langle n\boldsymbol{k}\middle|[r^a_i,p^b]\middle|m\boldsymbol{k}'\right\rangle + \left\langle n\boldsymbol{k}\middle|[r^a_e,p^b]\middle|m\boldsymbol{k}'\right\rangle = 0$ again, we can obtain

$$m\delta\left(\boldsymbol{k}-\boldsymbol{k}'\right)\omega_{nm}(r^b_{nm})_{;k^a} - m\delta\left(\boldsymbol{k}-\boldsymbol{k}'\right)r^b_{nm}\Delta^a_{mn}$$
$$= m\delta(\boldsymbol{k}-\boldsymbol{k}')\left(r^a_{nm}\Delta^b_{mn} + i\sum_{l,l\neq m\neq n}\left(\omega_{lm}r^a_{nl}r^b_{lm} - \omega_{nl}r^a_{lm}r^b_{nl}\right)\right). \quad (5)$$

By sorting it, the expansion of the generalized derivative of matrix elements shows as

$$(r^b_{nm})_{;k^a} = \frac{r^a_{nm}\Delta^b_{mn} + r^b_{nm}\Delta^a_{mn}}{\omega_{nm}} + \frac{i}{\omega_{nm}}\sum_l\left(\omega_{lm}r^a_{nl}r^b_{lm} - \omega_{nl}r^a_{lm}r^b_{nl}\right), \quad (6)$$

which is interpreted as a "sum rule". Here, $\Delta_{mn} = \frac{\boldsymbol{p}_{mm} - \boldsymbol{p}_{nn}}{m} = \frac{\partial\omega_{mn}}{\partial\boldsymbol{k}}$, represents the difference of group velocity of electrons.

Supplementary Table 1. Experimental crystallographic data for $NbOCl_2$ at ambient conditions obtained by single-crystal X-ray diffraction.

	$NbOCl_2$, Z=4	$NbOCl_2$, Z=8
Chemical formula	$NbOCl_2$	$NbOCl_2$
M_r	179.81	179.81
Crystal system, space group	Monoclinic, *C*2	Monoclinic, *C*2
a, b, c (Å)	12.8412(15), 3.9025(4), 6.7148(9)	12.8452(17), 3.9028(4), 13.4311(19)
α, β, γ (°)	90, 105.830(5), 90	90, 105.846(5), 90
V (Å^3)	323.74(7)	647.74(14)
Z	4	8
Density (Mg/m^3)	3.689	3.688
Wavelength (Å)	0.71073	0.71073
μ (mm^{-1})	5.089	5.087
Absorption correction	Multi-scan	Multi-scan
T_{min}, T_{max}	0.62, 0.85	0.60, 0.85
No. of measured, independent and observed reflections	575, 575, 544	1028, 1028, 677
R_{int}	0.036	0.104
θ_{max} (°)	31.00	29.57
Refinement on	F^2	F^2
$R[F^2>2\sigma(F^2)]$, $wR(F^2)$, S	0.034, 0.77, 1.10	0.057, 0.109, 1.16
Data / restraints / parameters	575/ 1/ 37	1028/ 1/ 73
Weighting scheme	$w=1/[\sigma^2(F_o^2)+(0.021P)^2+6.255P]$, where $P=(F_o^2+2F_c^2)/3$	$w=1/[\sigma^2(F_o^2)+(0.036P)^2+25.272P]$, where $P=(F_o^2+2F_c^2)/3$
$\Delta\rho_{max}$, $\Delta\rho_{min}$ (e Å-3)	2.22, -1.66	3.33, -3.08

Supplementary Table 2. Experimental structure refinement of $NbOCl_2$ at 5.7 GPa.

Empirical formula	$NbOCl_2$
Formula weight	179.81
Temperature	296 K
Wavelength	0.71073 Å
Crystal system	Monoclinic
Space group	*C*2/*c*
Unit cell dimensions	a = 11.699(7) Å, α = 90.00°<br>b = 3.8277(6) Å, β = 106.83(3)°<br>c = 13.157(3) Å, γ = 90.00°
Volume	563.3(4) $Å^3$
Z	8
Density (calculated)	4.236 g/cm^3
Absorption coefficient	5.84 mm^{-1}
F(000)	664
Crystal size	0.035 x 0.034 x 0.023 mm^3
θ range for data collection	3.2 to 25.3°
Index ranges	-6<=h<=6, -4<=k<=4, -14<=l<=14
Reflections collected	749
Independent reflections	147 [R(int) = 0.042]
Coverage of independent reflections	28.4%
Refinement method	Full-matrix least-squares on F^2
Data / restraints / parameters	147 / 24 / 37
Goodness-of-fit	1.24
Final R indices [>2σ(I)]	R_{obs} = 0.089, wR_{obs} = 0.227
R indices [all data]	R_{all} = 0.096, wR_{all} = 0.242
Largest diff. peak and hole	1.69 and -1.24 $e \cdot Å^{-3}$

$R = \Sigma||F_o|-|F_c|| / \Sigma|F_o|$, $wR = \{\Sigma[w(|F_o|^2 - |F_c|^2)^2] / \Sigma[w(|F_o|^4)]\}^{1/2}$ and calc

$w = 1/[\sigma^2(Fo^2)+(0.0503P)^2+0.0000P]$ where $P = (Fo^2+2Fc^2)/3$

Supplementary Table 3. Experimental atomic coordinates and equivalent isotropic atomic displacement parameters ($Å^2$) of $NbOCl_2$ at 5.7 GPa.

Label	x	y	z	Occupancy	U_{eq}^*
Nb1	0.5003 (6)	0.9513 (7)	0.6381 (3)	1	0.026 (4)
O1	0.498 (5)	0.486 (6)	0.6348 (17)	1	0.024 (7)
Cl1	0.677 (3)	0.994 (2)	0.7971 (9)	1	0.037 (5)
Cl2	0.345 (2)	0.001 (2)	0.4610 (8)	1	0.032 (5)

*U_{eq} is defined as one third of the trace of the orthogonalized U_{ij} tensor.

Supplementary Table 4. Experimental anisotropic atomic displacement parameters (Å^2) of $NbOCl_2$ at 5.7 GPa.

Label	U_{11}	U_{22}	U_{33}	U_{12}	U_{13}	U_{23}
Nb1	0.048 (12)	0.010 (2)	0.026 (4)	0.0001 (16)	0.019 (6)	-0.0037 (9)
O1	0.026 (15)	0.017 (7)	0.028 (10)	0.004 (9)	0.005 (10)	-0.001 (5)
Cl1	0.040 (13)	0.060 (4)	0.012 (7)	-0.007 (7)	0.008 (8)	0.000 (3)
Cl2	0.036 (14)	0.053 (4)	0.005 (6)	-0.007 (6)	0.003 (8)	-0.005 (3)

The anisotropic displacement factor exponent takes the form: $-2\pi^2[h^2a^{*2}U_{11} + \ldots + 2hkb^*c^*U_{23}]$.

Supplementary Table 5. Experimental bond lengths (Å) of $NbOCl_2$ at 5.7 GPa.

Label	Distances	Label	Distances
Nb1—O1	1.78 (2)	Nb1—Cl2	2.515 (18)
Nb1—O1^{i}	2.05 (2)	Nb1—Cl2^{iii}	2.53 (2)
Nb1—Cl1^{ii}	2.47 (3)	Nb1—Nb1^{ii}	2.947 (6)
Nb1—Cl1	2.48 (3)		

Symmetry codes: (i) *x*, *y*−1, *z*; (ii) −*x*+1, *y*, −*z*+3/2; (iii) −*x*+1, −*y*, −*z*+1; (iv) *x*, *y*+1, *z*.

Supplementary Table 6. Experimental bond angles (°) of $NbOCl_2$ at 5.7 GPa.

Label	Angles	Label	Angles
O1—Nb1—O1^{i}	175 (3)	Cl1^{ii}—Nb1—Cl2^{iii}	167.0 (7)
O1—Nb1—Cl1^{ii}	92.8 (17)	Cl1—Nb1—Cl2^{iii}	84.3 (12)
O1^{i}—Nb1—Cl1^{ii}	85.7 (14)	Cl2—Nb1—Cl2^{iii}	86.4 (12)
O1—Nb1—Cl1	96.2 (16)	O1—Nb1—Nb1^{ii}	91.6 (9)
O1^{i}—Nb1—Cl1	88.8 (15)	O1^{i}—Nb1—Nb1^{ii}	91.4 (8)
Cl1^{ii}—Nb1—Cl1	105.3 (13)	Cl1^{ii}—Nb1—Nb1^{ii}	53.0 (7)
O1—Nb1—Cl2	91.1 (15)	Cl1—Nb1—Nb1^{ii}	52.7 (7)
O1^{i}—Nb1—Cl2	84.0 (14)	Cl2—Nb1—Nb1^{ii}	136.0 (7)
Cl1^{ii}—Nb1—Cl2	83.0 (11)	Cl2^{iii}—Nb1—Nb1^{ii}	137.0 (9)
Cl1—Nb1—Cl2	168.6 (6)	Nb1^{ii}—Cl1—Nb1	74.3 (12)
O1—Nb1—Cl2^{iii}	94.9 (14)	Nb1—Cl2—Nb1^{iii}	93.6 (12)
O1^{i}—Nb1—Cl2^{iii}	85.7 (13)	Nb1—O1—Nb1^{iv}	175 (3)

Symmetry codes: (i) *x*, *y*−1, *z*; (ii) −*x*+1, *y*, −*z*+3/2; (iii) −*x*+1, −*y*, −*z*+1; (iv) *x*, *y*+1, *z*.

Supplementary Table 7. Experimental structure refinement of $NbOI_2$ at ambient condition.

Empirical formula	$NbOI_2$	
Formula weight	362.71	
Temperature	296(2) K	
Wavelength	0.71073 Å	
Crystal system	Monoclinic	
Space group	*C*2	
Unit cell dimensions	a = 15.113(3) Å	α = 90°
	b = 3.9389(8) Å	β = 103.845(5)°
	c = 7.5284(16) Å	γ = 90°
Volume	435.13(16) $Å^3$	
Z	4	
Density (calculated)	5.537 Mg/m^3	
Absorption coefficient	16.751 mm^{-1}	
F(000)	620	
Crystal size	0.040 x 0.035 x 0.032 mm^3	
θ range for data collection	2.78 to 25.66°	
Index ranges	-18<=h<=18, -4<=k<=4, -9<=l<=9	
Reflections collected	1971	
Independent reflections	797 [R(int) = 0.0358]	
Coverage of independent reflections	98.8 %	
Refinement method	Full-matrix least-squares on F^2	
Data / restraints / parameters	797 / 7 / 38	
Goodness-of-fit	1.094	
Final R indices [>2σ(I)]	R_1 = 0.0727, wR_2 = 0.1380	
R indices [all data]	R_1 = 0.0899, wR_2 = 0.1512	
Largest diff. peak and hole	4.680 and -4.540 $e·Å^{-3}$	

$R = \Sigma||F_o|-|F_c|| / \Sigma|F_o|$, $wR = \{\Sigma[w(|F_o|^2 - |F_c|^2)^2] / \Sigma[w(|F_o|^4)]\}^{1/2}$ and calc

$w = 1/[\sigma^2(Fo^2) + 146.4739P]$ where $P = (Fo^2+2Fc^2)/3$

Supplementary Table 8. Experimental atomic coordinates and equivalent isotropic atomic displacement parameters ($Å^2$) of $NbOI_2$ at ambient condition.

Label	x	y	z	Occupancy	Site	U_{eq}*
I001	0.6499 (2)	0.2350 (10)	0.0713 (5)	1	4c	0.0282 (8)
I002	0.6335 (2)	0.2326 (9)	0.5605 (5)	1	4c	0.0286 (8)
Nb03	0.5005 (3)	0.2852 (8)	0.2215 (9)	1	4c	0.0384 (14)
O004	0.500 (2)	0.751 (11)	0.221 (4)	1	4c	0.037 (8)

*U_{eq} is defined as one third of the trace of the orthogonalized U_{ij} tensor.

Supplementary Table 9. Experimental anisotropic atomic displacement parameters (Å^2) of $NbOI_2$ at ambient condition.

Label	U_{11}	U_{22}	U_{33}	U_{12}	U_{13}	U_{23}
I001	0.0483 (18)	0.0131 (14)	0.0274 (16)	0.001 (2)	0.0175 (15)	−0.001 (2)
I002	0.0534 (19)	0.0135 (15)	0.0176 (14)	0.003 (3)	0.0057 (15)	−0.001 (2)
Nb03	0.0193 (18)	0.007 (2)	0.086 (4)	0.003 (2)	0.007 (3)	0.004 (3)
O004	0.027 (14)	0.023 (14)	0.06 (2)	0.01 (2)	0.002 (16)	0.01 (2)

The anisotropic displacement factor exponent takes the form: $-2\pi^2[h^2a^{*2}U_{11} + ... + 2hka^*b^*U_{12}]$.

Supplementary Table 10. Experimental bond lengths (Å) of $NbOI_2$ at ambient condition.

Label	Distances	Label	Distances
I001—Nb03	2.764 (6)	Nb03—O004	1.83 (5)
I001—Nb03^{i}	2.769 (7)	Nb03—O004^{iii}	2.11 (5)
I002—Nb03	2.854 (7)	Nb03—Nb03^{i}	3.331 (14)
I002—Nb03^{ii}	2.903 (7)		

Symmetry codes: (i) −x+1, y, −z; (ii) −x+1, y, −z+1; (iii) x, y−1, z; (iv) x, y+1, z.

Supplementary Table 11. Experimental bond angles (°) of $NbOI_2$ at ambient condition.

Label	Angles	Label	Angles
Nb03—I001—Nb03^{i}	74.0 (2)	O004—Nb03—I002^{ii}	93.8 (11)
Nb03—I002—Nb03^{ii}	93.6 (2)	O004^{iii}—Nb03—I002^{ii}	85.7 (9)
O004—Nb03—O004^{iii}	179.2 (19)	I001—Nb03—I002^{ii}	167.1 (2)
O004—Nb03—I001	94.4 (11)	I001^{i}—Nb03—I002^{ii}	83.93 (16)
O004^{iii}—Nb03—I001	86.2 (9)	I002—Nb03—I002^{ii}	85.8 (2)
O004—Nb03—I001^{i}	93.7 (10)	O004—Nb03—Nb03^{i}	90.0 (11)
O004^{iii}—Nb03—I001^{i}	85.6 (9)	O004^{iii}—Nb03—Nb03^{i}	90.0 (9)
I001—Nb03—I001^{i}	105.4 (2)	I001—Nb03—Nb03^{i}	53.05 (17)
O004—Nb03—I002	94.5 (10)	I001^{i}—Nb03—Nb03^{i}	52.93 (19)
O004^{iii}—Nb03—I002	86.1 (9)	I002—Nb03—Nb03^{i}	136.8 (3)
I001—Nb03—I002	83.73 (16)	I002^{ii}—Nb03—Nb03^{i}	136.9 (3)
I001^{i}—Nb03—I002	167.2 (2)	Nb03—O004—Nb03^{iv}	179.2 (19)

Symmetry codes: (i) −x+1, y, −z; (ii) −x+1, y, −z+1; (iii) x, y−1, z; (iv) x, y+1, z.

Supplementary Table 12. Experimental structure refinement of $NbOI_2$ at 10.7 GPa.

Empirical formula	$NbOI_2$
Formula weight	362.71
Temperature	296.15(10) K
Wavelength	0.56087 Å
Crystal system	Monoclinic
Space group	*C*2/*m*
Unit cell dimensions	a = 13.27(7) Å α = 90°<br>b = 3.7721(11) Å β = 105.6(3)°<br>c = 7.222(6) Å γ = 90°
Volume	348.1(19) $Å^3$
Z	4
Density (calculated)	6.921 Mg/m^3
Absorption coefficient	19.742 mm^{-1}
F(000)	620
Crystal size	0.033 x 0.032 x 0.028 mm^3
θ range for data collection	2.310 to 21.204°
Index ranges	-7<=h<=6, -4<=k<=4, -9<=l<=9
Reflections collected	570
Independent reflections	124 [R(int) = 0.0851]
Coverage of independent reflections	29.8 %
Refinement method	Full-matrix least-squares on F^2
Data / restraints / parameters	124 / 24 / 26
Goodness-of-fit	1.098
Final R indices [>2σ(I)]	R_1 = 0.0937, wR_2 = 0.2260
R indices [all data]	R_1 = 0.1040, wR_2 = 0.2397
Largest diff. peak and hole	4.592 and -2.755 e·$Å^{-3}$

$R = \Sigma||F_o|-|F_c|| / \Sigma|F_o|$, $wR = \{\Sigma[w(|F_o|^2 - |F_c|^2)^2] / \Sigma[w(|F_o|^4)]\}^{1/2}$ and calc $w=1/[\sigma^2(Fo^2)+(0.2P)^2]$ where $P=(Fo^2+2Fc^2)/3$

Supplementary Table 13. Atomic coordinates and equivalent isotropic atomic displacement parameters ($Å^2$) of $NbOI_2$ at 10.7 GPa.

Label	x	y	z	Occupancy	Site	U_{eq}*
I001	0.3550 (10)	1.000000	0.9348 (6)	1	4i	0.012 (3)
I002	0.3260 (10)	1.000000	0.4010 (10)	1	4i	0.012 (3)
Nb03	0.4964 (13)	1.000000	0.7093 (10)	1	4i	0.010 (2)
O004	0.498 (10)	0.500000	0.723 (7)	1	4i	0.030 (18)

*U_{eq} is defined as one third of the trace of the orthogonalized U_{ij} tensor.

Supplementary Table 14. Anisotropic atomic displacement parameters (Å^2) of $NbOI_2$ at 10.7 GPa.

Label	U_{11}	U_{22}	U_{33}	U_{12}	U_{13}	U_{23}
I001	0.014 (9)	0.005 (2)	0.015 (3)	0.000	−0.002 (4)	0.000
I002	0.013 (9)	0.003 (2)	0.018 (2)	0.000	0.000 (4)	0.000
Nb03	0.010 (3)	0.006 (2)	0.014 (2)	0.000	0.002 (2)	0.000
O004	0.03 (4)	0.02 (2)	0.05 (2)	0.000	0.03 (3)	0.000

The anisotropic displacement factor exponent takes the form: $-2\pi^2[h^2a^{*2}U_{11} + ... + 2hka^*b^*U_{12}]$.

Supplementary Table 15. Bond lengths (Å) of $NbOI_2$ at 10.7 GPa.

Label	Distances	Label	Distances
I001—Nb03i	2.788 (15)	Nb03—Nb03ii	3.050 (10)
I001—Nb03	2.795 (18)	Nb03—O004iii	1.889 (2)
I002—Nb03ii	2.68 (2)	Nb03—O004	1.889 (2)
I002—Nb03	2.714 (18)		

Symmetry codes: (i) −x+1, −y+2, −z+2; (ii) −x+1, −y+2, −z+1; (iii) x, y+1, z; (iv) x, y−1, *z*.

Supplementary Table 16. Bond angles (°) of $NbOI_2$ at 10.7 GPa.

Label	Angles	Label	Angles
Nb03^{i}—I001—Nb03	96.8 (6)	O004^{iii}—Nb03—I001^{i}	87 (3)
Nb03^{ii}—I002—Nb03	68.8 (4)	O004^{iii}—Nb03—I001	88 (3)
I001^{i}—Nb03—I001	83.2 (6)	O004—Nb03—I001^{i}	87 (3)
I001—Nb03—Nb03^{ii}	141.5 (10)	O004—Nb03—I001	88 (3)
I001^{i}—Nb03—Nb03^{ii}	135.3 (9)	O004^{iii}—Nb03—I002^{ii}	91 (3)
I002—Nb03—I001^{i}	169.6 (5)	O004—Nb03—I002	92 (3)
I002—Nb03—I001	86.4 (7)	O004—Nb03—I002^{ii}	91 (3)
I002^{ii}—Nb03—I001	162.5 (7)	O004^{iii}—Nb03—I002	92 (3)
I002^{ii}—Nb03—I001^{i}	79.2 (6)	O004—Nb03—Nb03^{ii}	92.9 (13)
I002^{ii}—Nb03—I002	111.2 (4)	O004^{iii}—Nb03—Nb03^{ii}	92.9 (13)
I002—Nb03—Nb03^{ii}	55.1 (6)	O004^{iii}—Nb03—O004	174 (3)
I002^{ii}—Nb03—Nb03^{ii}	56.1 (5)	Nb03^{iv}—O004—Nb03	174 (3)

Symmetry codes: (i) −x+1, −y+2, −z+2; (ii) −x+1, −y+2, −z+1; (iii) x, y+1, z; (iv) x, y−1, z.

Supplementary Table 17. Experimental structure refinement of $NbOCl_2$ at 500 K.

Empirical formula	$NbOCl_2$
Formula weight	179.81
Temperature	500 K
Wavelength	0.71073 Å
Crystal system	Monoclinic
Space group	*C*2/*c*
Unit cell dimensions	a = 12.9128(15) Å, α = 90.00°<br>b = 3.8993(5) Å, β = 105.295(5)°<br>c = 13.4507(17) Å, γ = 90.00°
Volume	653.27(14) $Å^3$
Z	8
Density (calculated)	3.656 g/cm^3
Absorption coefficient	5.04 mm^{-1}
F(000)	664
Crystal size	0.040 x 0.034 x 0.033 mm^3
θ range for data collection	3.1 to 27.9°
Index ranges	-16<=h<=16, -5<=k<=5, -17<=l<=17
Reflections collected	2493
Independent reflections	772 [R(int) = 0.058]
Coverage of independent reflections	99.0%
Refinement method	Full-matrix least-squares on F^2
Data / restraints / parameters	772 / 0 / 37
Goodness-of-fit	1.159
Final R indices [>2σ(I)]	R_{obs} = 0.078, wR_{obs} = 0.160
R indices [all data]	R_{all} = 0.103, wR_{all} = 0.171
Largest diff. peak and hole	1.834 and -2.217 $e{\cdot}Å^{-3}$

$R = \Sigma||F_o|-|F_c|| / \Sigma|F_o|$, $wR = \{\Sigma[w(|F_o|^2 - |F_c|^2)^2] / \Sigma[w(|F_o|^4)]\}^{1/2}$ and calc
$w = 1/[\sigma^2(Fo^2)+(0.0725P)^2+26.6676P]$ where $P = (Fo^2+2Fc^2)/3$

Supplementary Table 18. Experimental atomic coordinates and equivalent isotropic atomic displacement parameters ($Å^2$) of $NbOCl_2$ at 500 K.

Label	x	y	z	Occupancy	U_{eq}*
Nb1	0.49955 (8)	0.0561 (3)	0.36095 (9)	1	0.0192 (4)
O3	0.4984 (7)	0.513 (2)	0.3631 (7)	1	0.024 (2)
Cl1	0.6346 (3)	−0.0006 (9)	0.5334 (2)	1	0.0283 (8)
Cl2	0.3459 (3)	0.0076 (9)	0.2107 (2)	1	0.0287 (8)

*U_{eq} is defined as one third of the trace of the orthogonalized U_{ij} tensor.

Supplementary Table 19. Experimental anisotropic atomic displacement parameters (Å^2) of $NbOCl_2$ at 500 K.

Label	U_{11}	U_{22}	U_{33}	U_{12}	U_{13}	U_{23}
Nb1	0.0279 (6)	0.0110 (6)	0.0196 (6)	0.0004 (5)	0.0077 (4)	0.0001 (5)
O3	0.041 (5)	0.009 (5)	0.028 (5)	0.003 (4)	0.018 (4)	0.005 (5)
Cl1	0.0304 (16)	0.034 (2)	0.0201 (15)	0.0001 (13)	0.0061 (12)	0.0018 (15)
Cl2	0.0290 (15)	0.033 (2)	0.0252 (16)	−0.0009 (13)	0.0099 (13)	−0.0007 (15)

The anisotropic displacement factor exponent takes the form: $-2\pi^2[h^2a^{*2}U_{11} + \ldots + 2hkb^*c^*U_{23}]$.

Supplementary Table 20. Experimental bond lengths (Å) of $NbOCl_2$ at 500 K.

Label	Distances	Label	Distances
Nb1—O3	1.781 (8)	Nb1—Cl1	2.517 (3)
Nb1—O3^{i}	2.119 (8)	Nb1—Cl1^{iii}	2.522 (3)
Nb1—Cl2	2.438 (3)	Nb1—Nb1^{ii}	2.988 (2)
Nb1—Cl2^{ii}	2.441 (3)		

Symmetry codes: (i) $x, y-1, z$; (ii) $-x+1, y, -z+1/2$; (iii) $-x+1, -y, -z+1$; (iv) $x, y+1, z$.

Supplementary Table 21. Experimental bond angles (°) of $NbOCl_2$ at 500 K.

Label	Angles	Label	Angles
O3—Nb1—O3^{i}	177.9 (5)	Cl2—Nb1—Cl1^{iii}	86.04 (11)
O3—Nb1—Cl2	94.8 (3)	Cl2^{ii}—Nb1—Cl1^{iii}	165.86 (12)
O3^{i}—Nb1—Cl2	85.8 (3)	Cl1—Nb1—Cl1^{iii}	83.39 (11)
O3—Nb1—Cl2^{ii}	95.5 (3)	O3—Nb1—Nb1^{ii}	91.1 (3)
O3^{i}—Nb1—Cl2^{ii}	86.4 (2)	O3^{i}—Nb1—Nb1^{ii}	90.9 (2)
Cl2—Nb1—Cl2^{ii}	103.77 (11)	Cl2—Nb1—Nb1^{ii}	52.29 (8)
O3—Nb1—Cl1	94.6 (3)	Cl2^{ii}—Nb1—Nb1^{ii}	52.18 (8)
O3^{i}—Nb1—Cl1	84.5 (3)	Cl1—Nb1—Nb1^{ii}	137.42 (10)
Cl2—Nb1—Cl1	166.34 (12)	Cl1^{iii}—Nb1—Nb1^{ii}	138.32 (9)
Cl2^{ii}—Nb1—Cl1	85.26 (11)	Nb1—Cl1—Nb1^{iii}	96.61 (11)
O3—Nb1—Cl1^{iii}	93.8 (3)	Nb1—Cl2—Nb1^{ii}	75.52 (11)
O3^{i}—Nb1—Cl1^{iii}	84.2 (2)	Nb1—O3—Nb1^{iv}	177.9 (5)

Symmetry codes: (i) $x, y-1, z$; (ii) $-x+1, y, -z+1/2$; (iii) $-x+1, -y, -z+1$; (iv) $x, y+1, z$.

References

[1] Schäfer H, Schnering H G. Metall-Metall-Bindungen bei niederen Halogeniden, Oxyden und Oxydhalogeniden schwerer Übergangsmetalle Thermochemische und strukturelle Prinzipien. Angewandte Chemie 1964, 76, 833-849.

[2] Guo, Q.; Qi, X.-Z.; Zhang, L.; Gao, M.; Hu, S.; Zhou, W.; Zang, W.; Zhao, X.; Wang, J.; Yan, B.; Xu, M.; Wu, Y.-K.; Eda, G.; Xiao, Z.; Yang, S. A.; Gou, H.; Feng, Y. P.; Guo, G.-C.; Zhou, W.; Ren, X.-F.; Qiu, C.-W.; Pennycook, S. J.; Wee, A. T. S. Ultrathin quantum light source with van der Waals $NbOCl_2$ crystal. Nature 2023, 613, 53−59.
